# A potential protective role of losartan against coronavirus-induced lung damage

**DOI:** 10.1017/ice.2020.80

**Published:** 2020-03-17

**Authors:** Mehrdad Zeinalian, Azhar Salari-Jazi, Amin Jannesari, Hossein Khanahmad

**Affiliations:** 1 Department of Genetics and Molecular Biology, School of Medicine, Isfahan University of Medical Sciences, Isfahan, Iran; 2 Department of Microbiology, School of Medicine, Isfahan University of Medical Sciences, Isfahan, Iran; 3 School of Pharmacy and Pharmaceutical Sciences, Isfahan University of Medical Sciences, Isfahan, Iran

*To the Editor—*Currently, the coronavirus pandemic imposes a growing general panic worldwide. Millions of people are affected daily by this virus and thousands have already died around the world. The COVID-19 disease is caused by SARS-CoV-2, a novel variant of the virus, similar SARS-CoV. SARS-CoV-2 is a β-genus coronavirus that belongs to a large family of single-stranded enveloped RNA viruses.^1^

After entering the body, coronaviruses fuse their envelopes with the membranes of host cells, then they transport their genetic material into the affected cells. This essential fusion is mediated by glycosylated spike proteins on the surface of the virion interacting with proper surface receptors on the membrane of the host cell. Angiotensin-converting enzyme 2 (ACE2) receptor is a known human cell-surface protein to which CoV spike proteins specifically bind.^2^

ACE2 is a vital protein in the renin–angiotensin system (RAS). The activation of RAS is triggered by the secretion of renin from the kidney, through juxtaglomerular cells. Renin is a protease that cleaves angiotensinogen, the precursor of angiotensin, which is made by the liver; it produces an inactive peptide, angiotensin I (AngI). ACE then mediates the conversion of AngI to AngII, a major RAS effector. ACE is a protein that is highly expressed on membranes of vascular endothelial cells, predominantly in lung tissue.^3^ Most RAS-associated physiologic effects are driven by the interaction of AngII with a G-protein coupled AngII type 1 (AT1) receptor. This activates a physiologic pathway in different systems: kidney, liver, central nervous system, respiratory system, and/or cardiovascular system. Some crucial events are regulated via active AT1 receptors including arterial pressure, fluid and sodium balance, fibrosis, and cellular growth and migration.^2^

Some studies have reported an increased inflammatory responses due to AT1 activated by AngII.^4^ In some pathological conditions, overactivation of AT1 may lead to damaging events such as fibrosis in different organs (eg, liver and lungs), perhaps through increasing TGF-β expression.^4^ Other studies have indicated that ACE2 has a protective effect on the fibrogenesis and inflammation of different organs, as well as the liver and the lungs.^4,5^ Taking these studies together, the ACE–AngII–AT1 axis in the RAS system shows a predominant role in organ fibrosis, particularly in the lungs and liver.^4,5^

According to some recent studies, ACE2 has a regulatory effect on innate immunity and gut microbiota composition.^6^ Moreover, ACE2 has a determinant antifibrotic role in the lung injury induced by sepsis, acid aspiration, SARS, and lethal avian influenza A H5N1 virus.^6^

On the other hand, the most common complication leading to the COVID-19–induced mortality is respiratory failure due to extensive, accelerating lung fibrogenesis. Rather than PCR-based testing to detect CoV infection, a radiologic lung infiltration pattern on chest X ray could have diagnostic value in screening patients suspected of COVID-19.^7,8^ The cytopathic effects of SARS-CoV-2 due to its massive replication in infected cells, need more time than the acute manifestation of COVID-19. Thus, the acute acceleration of lung fibrosis in COVID-19 can be explained by ACE–AngII–AT1 overactivation caused by the SARS-CoV-2 virus.^8^

Losartan is an AT_1_ antagonist with a selective, competitive function that decreases the end-organ responses to AngII. This common antihypertensive agent is currently prescribed to high-blood-pressure patients, particularly those who are prone to diabetic nephropathies.^9^

Losartan counteracts the physiological effects of AngII, including release of aldosterone. Plasma renin activity then increases because of the absence of AngII feedback. Losartan induces several biochemical events: converting angiotensinogen to AngI and AngI to AngII (by ACE), and vasoconstriction and aldosterone release (by AngII). Aldosterone leads to the retention of sodium in the kidney, which increases the blood pressure. Losartan can neutralize the downstream effect of renin and AngII, ultimately resulting in lower blood pressure.^10^

According to some limited studies, losartan has an inhibitory effect on the development of liver fibrosis and even contributes to the regression of the fibrosis stage in chronic HCV patients.^11^ In another study, losartan led to the downregulation of TGF-β1 and fibrogenic molecules in human trabecular meshwork cells infected by cytomegalovirus. Thus, losartan has the potential to decrease trabecular meshwork fibrosis in patients with cytomegalovirus-induced hypertensive anterior uveitis.^12^ Recently, losartan has been suggested for the treatment of Marfan syndrome. Losartan reduces the TGF-β level and, consequently, fibrosis.^13^ Some experimental research has also confirmed the preventive effect of losartan against lung fibrosis in paraquat poisoning.^14^

Accordingly, losartan is a selective antagonist of AT1 receptor that exerts an inhibitory effect on the ACE–AngII–AT1 axis in the RAS system, a known molecular pathway for end-organ fibrosis. Thus, losartan may be suggested as a potential agent of protection from lung damage induced by COVID-19. Losartan may also have a protective function against lung fibrosis through other molecular mechanisms such as the downregulation of TGF-β1. This hypothesis need to be verified through in vitro and in vivo investigations.
